# Supplementary material for: On the Secondary Structure of Silk Fibroin Nanoparticles Obtained Using Ionic Liquids: An Infrared Spectroscopy Study
Source: Polymers (Basel). 2020 Jun 5;12(6):1294. doi: 10.3390/polym12061294 (PMC7361871; doi:10.3390/polym12061294)
Supplement: Supplementary file 1 [file polymers-12-01294-s001.pdf]

polymers

MDPI

Supplementary information

On the secondary structure of silk fibroin nanoparticles obtained using ionic liquids: An Infrared spectroscopy study

Guzmán Carissimi[1], Cesare M. Baronio[2], Mercedes G. Montalbán[1], Gloria Víllora[1], Andreas Barth[2*].

[1] Department of Chemical Engineering, Faculty of Chemistry, University of Murcia, Spain
[2] Department of Biochemistry and Biophysics, Stockholm University, Sweden
* Correspondence: barth@dbb.su.se

Received: date; Accepted: date; Published: date

EmimAc and EmimAc/Water Subtraction

To study the SF dissolved in EmimAc and EmimAc/water, we measured the spectra of the solutions and performed a subtraction to remove the solvent(s) absorption. This procedure can be highly sensitive to the subtraction factor used. For this reason, several subtraction factors were tested, the results are collected in Figure S1. For the [SF-EmimAc] solution, Figure S1d, it can be seen that the resultant amide I band is insensitive to the subtraction factor used. In the case of [SF-EmimAc/H_2O] solution, Figure S1e, the water absorption band at ca. 1655 falls in the amide I region, making the difference spectrum in that region sensitive to the subtraction factor. To overcome this problem, heavy water was used instead, where the amide region is almost unaffected by the subtraction factor used as shown in Figure S1f.

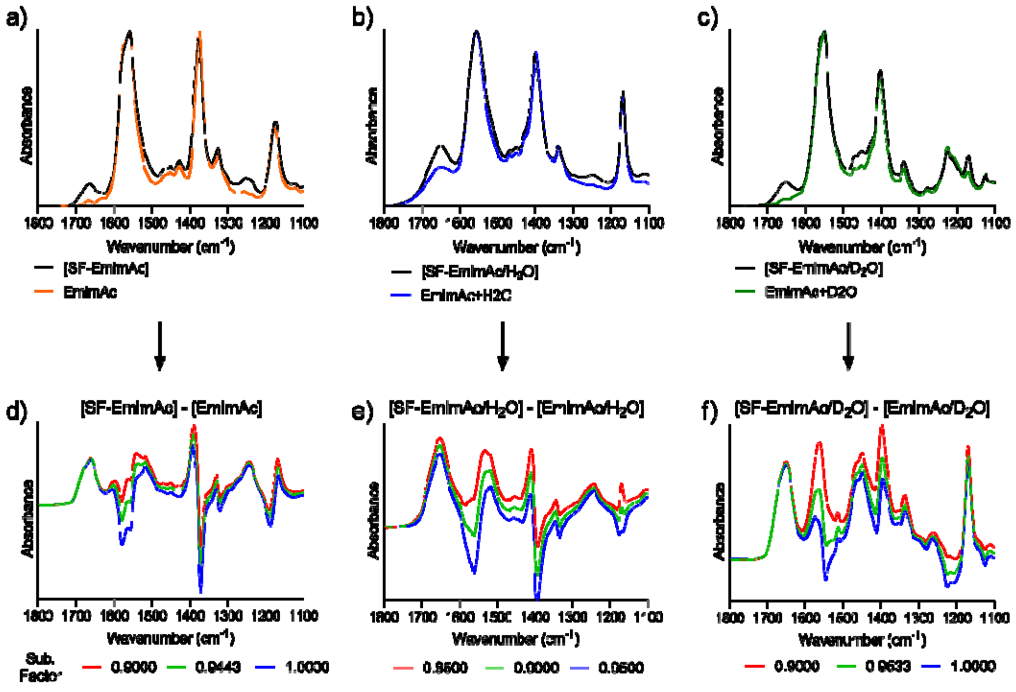

Figure S1. Top row: Silk fibroin solutions in a) EmimAc, b) EmimAc/H_2O and c) EmimAc/D_2O. Bottom Row: SF spectra after subtraction of solvent absorption d) EmimAc, e) EmimAc/H_2O and F) EmimAc/D_2O with different subtraction factors indicated in the figures.

© 2020 by the authors. Submitted for possible open access publication under the terms and conditions of the Creative Commons Attribution (CC BY) license (http://creativecommons.org/licenses/by/4.0/).
